# Editorial: Non-Invasive Measures of Cardiovascular Function and Health: Special Considerations for Assessing Lifestyle Behaviours

**DOI:** 10.3389/fcvm.2022.902883

**Published:** 2022-04-13

**Authors:** Simon Fryer, Daniel Credeur, Keeron Stone, Lee Stoner

**Affiliations:** ^1^School of Sport and Exercise, University of Gloucestershire, Gloucester, United Kingdom; ^2^Department of Nutrition and Exercise Physiology, University of Missouri, Columbia, MO, United States; ^3^Department of Epidemiology, Gillings School of Global Public Health, University of North Carolina at Chapel Hill, Chapel Hill, NC, United States

**Keywords:** sedentary, vascular–diagnosis, vascular function, arterial stiffness, biomarkers

In recent decades it has become clear that lifestyle behaviors such as diet (1), risky alcohol consumption (2), cigarette smoking (Wang et al.), sedentary behavior (3), and physical inactivity (4) are all factors contributing to cardiovascular disease (CVD) incidence and mortality. And, as cardiovascular diseases remain the largest cause of death in the Western world, gaining further knowledge of cardiovascular dysfunction in response to these lifestyle behaviors is of paramount importance. This Research Topic which includes 14 research papers (Figure 1), has helped further our knowledge in three important areas of lifestyle behaviors and cardiovascular physiology: First, it has highlighted the importance several modifiable, behavioral risk factors associated with CVD (El-Battrawy et al.; Wang et al.; Zuo et al.), second, it has expanded our understanding of a number of biomarkers and risk scores which are related to CVD (Bo et al.; Cang et al.; Hsu et al.; Tsai et al.; Ying et al.), third it helped us to better understand and interpret markers of arterial stiffness (Elliot et al.; Lane-Cordova and Bouknight; Stone et al.) and lastly, it has allowed us to provide guidance on how to assess cardiovascular function in response to a common and biologically novel lifestyle behavior, prolonged sitting (Stoner et al.). Collectively this new information is an important step forwards in improving our understanding of CVD, and allows us to work toward the development of public health policy aimed at reducing the CVD burden.

**Figure 1:**
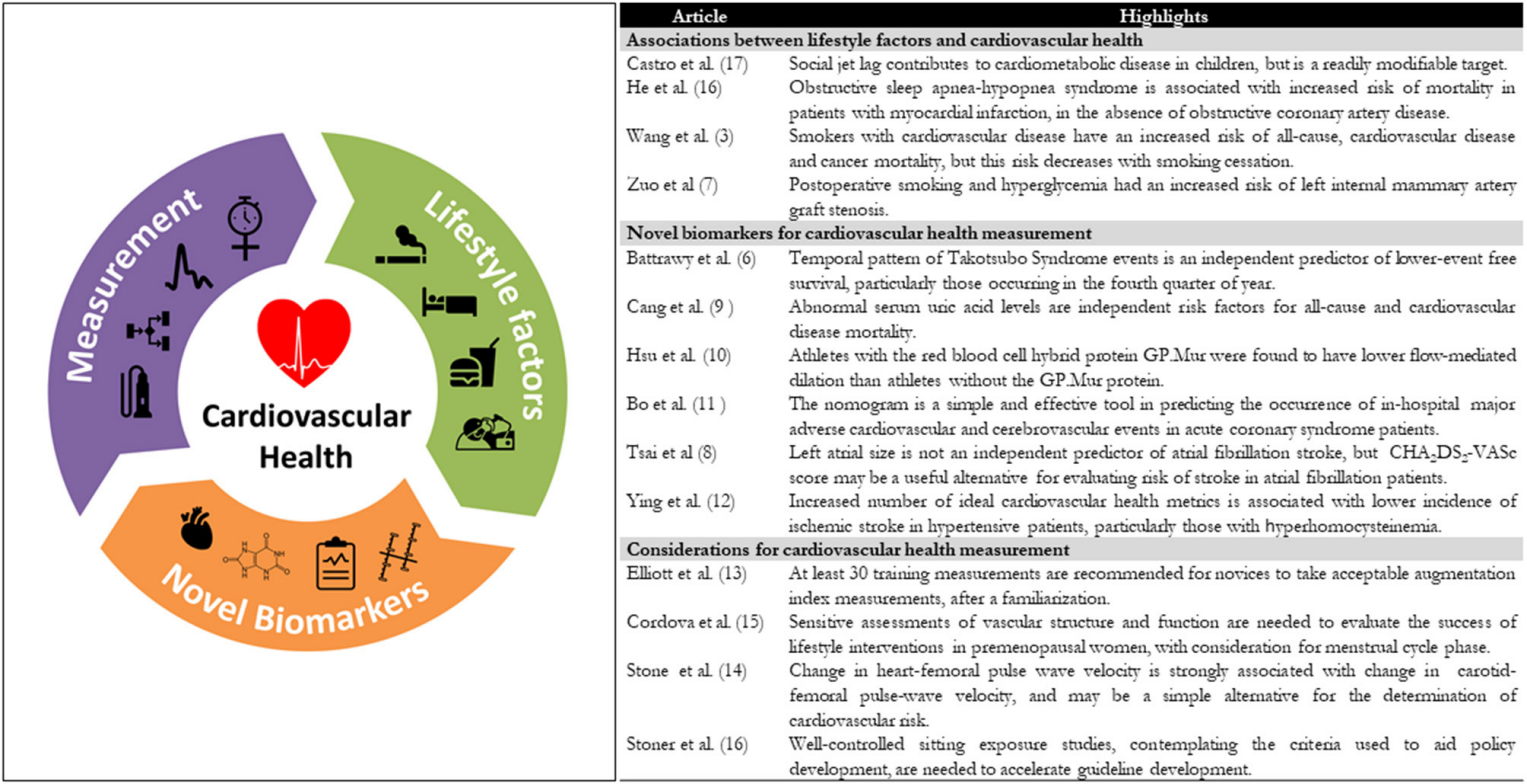
Study highlights for the non-invasive measures of cardiovascular function and health: special considerations for assessing lifestyle behaviors Research Topic.

In order to inform public health policy surrounding cardiovascular health and modifiable lifestyle behaviors, robust RCT trials need to be conducted (5). As such, simple but reliable non-invasive measures of assessing key markers of vascular health are needed. One key marker of vascular health, which predicts CVD and mortality is carotid-femoral pulse wave velocity (cfPWV) the gold standard measure of arterial stiffness (6). In our topic, Elliot et al. reported that in order to be able to conduct reliable cfPWV measurements, a novice operator needed a familiarization session, guided practice measurements on 5 people followed by measurements with >30 people; demonstrating the simplicity and novice operator reliability of this measure, its highlights the potential use of cfPWV in large RCTs and epidemiological studies. Stone et al. built upon this work by demonstrating that heart-femoral PWV (hfPWV), which is simpler to assess than cfPWV, and is less likely to be affected by subject-level factors such as carotid plaque, is strongly associated with cfPWV. As such, hfPWV may be an even simpler alternative to cfPWV in the identification of CVD risk in both clinical and epidemiological settings (Stone et al.). Assessments of PWV were part of the physiology toolbox developed by Stoner et al. The authors described the need for a set of study guidelines/considerations for assessing the physiological responses to acute sitting exposure, a novel and biologically distinct CVD risk factor (Stoner et al.). This work marks an important milestone in the need for the research community to improve cross-study methodological standardization.

In addition to non-invasive assessments of arterial function, this topic also collectively looked at a range of lifestyle and behavioral risk factors associated with CVD. Whilst some data reinforced evidence highlighting that the earlier and less an individual smoke, the lower the chances of premature death from CVD (Wang et al.), other research focused on more novel risk factors such as sleep apnea (He et al.). He et al. report an increased risk of mortality from obstructive sleep apnea-hypopnea syndrome in patients with myocardial infarction in the absence of obstructive coronary artery disease. Another novel predictor of cardiovascular health reported within this Research Topic, is social jetlag in children (Castro et al.). Castro et al. reported that social jetlag, defined as the difference between a person's social rhythms and circadian clock, is linked to both vascular health and cholesterol in preadolescent children. Whilst the authors did not determine biological paucity of this relationship, the findings are of great importance for the early stages of public health policy development.

This Research Topic has answered a number of prominent research questions surrounding non-invasive measures of cardiovascular function and health within the context of assessing lifestyle behaviors. However, it has highlighted several important gaps in the literature which warrant further research. For example, given that hfPWV is less likely than cfPWV to be affected by within subject factors such as the presence of carotid plaques, understanding whether it can be quickly and reliably taught in novices would help with justifying its use in the design of large cohort RCTs or epidemiological studies.

## Author Contributions

SF: design, writing, and topic editor. DC: proof reading and topic editor. KS: proof reading and figure construction. LS: design, proof reading, and topic editor. All authors contributed to the article and approved the submitted version.

## Conflict of Interest

The authors declare that the research was conducted in the absence of any commercial or financial relationships that could be construed as a potential conflict of interest.

## Publisher's Note

All claims expressed in this article are solely those of the authors and do not necessarily represent those of their affiliated organizations, or those of the publisher, the editors and the reviewers. Any product that may be evaluated in this article, or claim that may be made by its manufacturer, is not guaranteed or endorsed by the publisher.
